# How muscle synergies fail to solve the muscle redundancy problem during human reaching

**DOI:** 10.1101/2024.02.12.579990

**Published:** 2024-02-14

**Authors:** Anna S Korol, Valeriya Gritsenko

**Affiliations:** Department of Neuroscience, School of Medicine, West Virginia University, Morgantown, USA; Rockefeller Neuroscience Institute, West Virginia University, Morgantown, USA; Department of Human Performance, Division of Physical Therapy, School of Medicine, West Virginia University, Morgantown, USA; Department of Neuroscience, School of Medicine, West Virginia University, Morgantown, USA; Rockefeller Neuroscience Institute, West Virginia University, Morgantown, USA

**Keywords:** Muscle coactivation, EMG, gravitational torque component, dynamical torque component, active muscle torque, electromyography, neuromechanics, limb dynamics, hand dominance, gravity, postural forces

## Abstract

The production of movement involves integrating biomechanical, neural, and environmental factors. The biomechanics is complex enough that neural sensorimotor circuits must embed its dynamics for efficient and robust control. However, a problem of redundancy exists, i.e., the problem of choosing among multiple muscles and combinations of joint angles that are possible for a given desired hand position or motion. This problem may be resolved by reducing the dimensionality of the space of motor commands by the central nervous system, i.e., through muscle synergies or motor primitives. Other studies have obtained muscle synergies using decomposition methods. However, we posit that it is not sufficient to show the existence of a low dimensional space, one needs to demonstrate the utility of the obtained synergies in controlling movement. Here we defined a muscle synergy as a single control signal producing specific force direction. We then tested a hypothesis that such muscle synergies exist using dimensionality reduction method. Our approach takes advantage of the close relationship between the temporal profiles of muscle activity observed with electromyography and the joint moments they produce during reaching derived from motion capture. We recorded electromyography of 12 muscles and the kinematics of both arms in 14 right-handed participants performing reaching movements in multiple directions from different starting positions. We used principal component analysis to evaluate the contribution of individual muscles to supporting the arm against gravity and producing propulsive forces. Results show that the joint torques in specific directions (flexion or extension) required to move toward a target were not produced by consistent muscle groups in most conditions as would be expected from the muscle synergy definition outlined above. We further show that both agonistic and antagonistic muscles coactivate in flexible muscle groups but to a different extent between the dominant and non-dominant arms. Our main findings indicate that the nervous system solves the problem of choosing which muscles to activate and when by taking into account limb dynamics rather than reducing the dimensionality through muscle synergies. Furthermore, our data supports the idea of two neural controllers that target different muscle groups in the arm and hand for gross postural and fine goal-directed control of reaching.

## Introduction

The production of movement involves integrating biomechanical, neural, and environmental factors. Muscle recruitment by the central nervous system (CNS) causes motion or lack thereof, such as when maintaining posture, through the nonlinear production of forces by muscles about the degrees of freedom (DOFs) of the joints. Body inertia, the number of DOFs, and external forces then shape the resulting posture and movement. This biomechanics is complex enough that neural sensorimotor circuits must embed its dynamics for efficient and robust control [1–3]. However, a problem of redundancy exists, i.e., the problem of choosing among multiple muscles and combinations of joint angles that are possible for a given desired hand position or motion. This problem may be resolved by reducing the dimensionality of the space of motor commands by the CNS, i.e., through muscle synergies or motor primitives. This concept implies that muscles (or motoneurons in the spinal cord) are recruited in groups that represent a specific action so that a combination of the smaller number of synergies can produce a larger number of different movements [4–8]. The anatomical organization of muscles that form agonistic and antagonistic groups support the idea of modular control targets [9]. The spatial organization of motoneuron pools in the spinal cord also captures the functional relationships of the muscles they innervate, further supporting modularity [10]. Indeed, the neural activation of muscles observed with electromyography (EMG) is reducible to a low dimensional space for certain types of movements, including reaching [4,8,11]. However, we posit that it is not sufficient to show the existence of a low dimensional space, one needs to demonstrate the utility of the obtained synergies in controlling movement. For example, if a muscle synergy represents a given controlled force direction, then it needs to scale with the amount of that force across movements in different directions. For example, a single control signal can activate both brachioradialis and biceps muscles to produce elbow flexion moment. A decomposition method can identify this control signal as a synergy represented by the relative contribution of this synergy to each muscle activity profile for a given movement (scores) in a lower dimensional space. Assuming that this low dimensional space is aligned with the joint moments needed to produce these movements [12], then we expect the synergy scores to scale with the amplitude of the force that that synergy controls. We will test this idea in this study using EMG recorded during human center-out reaching movements.

The temporal profile of the envelope of surface EMG is closely related to the force the corresponding muscle is producing in response to neural activation [13]. More recently we have shown that for reaches with the dominant hand towards visual targets in three-dimensional space, the EMG profiles are closely related to the active joint moments that underly the movements [12]. Specifically, the static component of EMG that underlies postural forces needed to support the arm in specific position and during transitions between positions is closely related to the components of joint moments that include gravity terms in the equations of motion. This suggests that the static component of EMG envelope from a given muscle reflects the muscle’s contribution to counteracting gravity load on the joints it spans. Moreover, the residual phasic component of EMG is closely related to the residual joint moment that underlies the acceleration and deceleration forces toward the reaching goal after gravity-related component is subtracted. This suggests that the phasic component of EMG envelope from a given muscle reflects the muscle’s contribution to propulsion in a given direction. Here we will test the generalizability of these relationships between EMGs and joint moments to the non-dominant limb and across different workspaces.

In this study, healthy human participants performed unconstrained pointing movements toward visual targets located equidistantly from one of two central targets. This resulted in reaching movements in multiple directions performed by either dominant (right) or non-dominant (left) arm that spanned the typical reaching workspace. We collected motion capture and EMG data and ran dynamic simulations with individualized inertial models of the arm to compute joint moments and their components. We tested two hypotheses. The first hypothesis was that the muscle activity profiles reflect the forces needed to produce the movement. This hypothesis tests the generalizability of conclusions from Olesh et al. [12]. The second hypothesis was that muscle synergies captured with dimensionality reduction methods represent force directions controlled by the CNS.

## Methods

### Data Collection

The experimental protocol was approved by the Institutional Review Board of West Virginia University (Protocol #1311129283). The recruitment for this study started in March 1^st^, 2014 and ended on July 25^th^, 2016. Participants provided a written informed consent prior the start of experiments. A second member of the investigative team witnessed the signing of the consent form.

Fourteen healthy participants (mean ± std age: 35.43 ± 17.62 years, 6 females and 8 males) performed a modified center-out reaching task by pointing to visual targets in virtual reality (software Vizard by Worldviz, Oculus Rift) [12]. All participants reported they were right-handed and had no neurological or musculoskeletal conditions that could alter movement. Participants repeated reaching movements between a central target and one of 14 targets located along a sphere, with 8 targets placed equidistantly on a horizontal circle parallel with the floor and 8 targets placed equidistantly on a vertical circle parallel to the body sagittal plane, with 2 targets sharing locations in both circles as in Olesh *et al*. [12]. Participants were instructed to point to targets with the index finger, while holding their hand palm down, as quickly and accurately as they can without moving their trunk and wrist. The distances toward the targets were normalized for each subject based on their arm segment lengths to minimize the inter-subject variability in angular kinematics. Each movement to and from each target was repeated 15 times in a randomized order.

**Fig 1.**
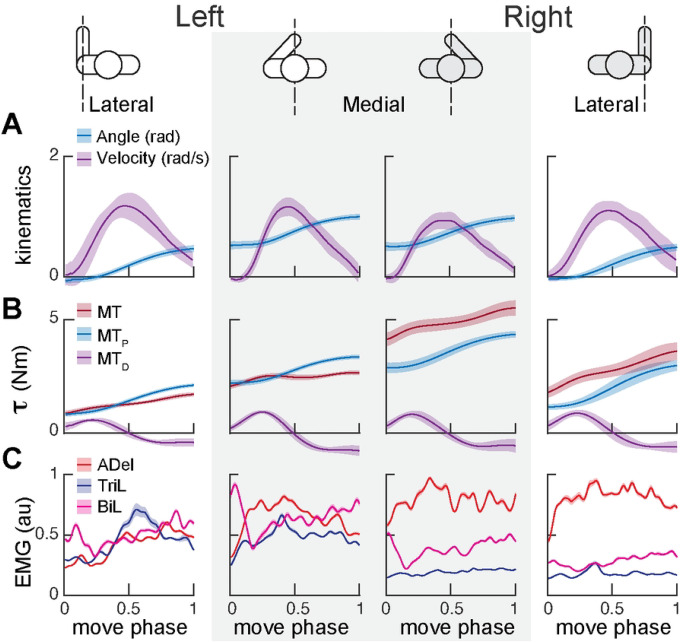
Examples of kinematics, dynamics, and muscle activity profiles during reaching. Each plot shows profiles per condition averaged across repetitions (n = 15) of the same reaching movement in one direction by one participant. (**A**) Profiles (solid lines) and standard deviation (shaded areas) of shoulder flexion-extension angle and angular velocity. (**B**) Shoulder flexion-extension torque (t) profiles that caused the movements in (**A**). (**C**) Normalized electromyography (EMG) profiles of anterior deltoid (ADel), triceps long (TriL), and biceps long (BiL) that accompanied the movements in (**A**). Shaded areas show the standard error for the mean across repetitions (n = 10) of the same movement.

The center-out and return movements were repeated with each arm starting at one of two locations of the central target. The central target was placed at the same distance relative to the participant’s shoulder scaled to their arm length. This further minimized the inter-subject variability in angular kinematics. In the lateral starting position, the central target was placed at a location that positioned the shoulder at 0 angle of all degrees of freedom and elbow at 90 degrees, so that the upper arm was parallel to the trunk and the forearm was parallel to the floor (Fig. 1, pictograms in top corners, view from above). In the medial starting position, the central target was placed at a location that positioned the hand at the midline of the body and at the same distance from the trunk as in the lateral starting position (Fig. 1, pictograms in top center, view from above). The medial starting position was the same for reaches with both right and left arms representing common medial workspace. Thus, four conditions were created based on the location of the central target and the arm used to make reaches, i.e., LLat and RLat for reaches by left and right arm respectively in their respective lateral workspaces and LMed and RMed for the reaches by left and right arm respectively in the common medial workspace.

Movement was recorded and visualized in virtual reality using the Impulse system (PhaseSpace Inc) at the temporal resolution of 480 Hz. We tracked the location of 8 light emitting diodes placed on the bony landmarks of the trunk, arm, and hand with 8 cameras as described in detail in Olesh et al. [12]. Motion capture and EMG data were imported into MATLAB (MathWorks Inc) and processed using custom scripts.

Surface electromyography (EMG) was recorded at the temporal resolution of 2,000 Hz using MA400–28 (MotionLab Systems). EMG was captured from 12 arm muscles: the clavicular head of pectoralis (Pec), teres major (TerM), anterior deltoid (ADel), posterior deltoid (PDel), the long and lateral heads of triceps (TriL and TriS), the short and long heads of biceps (BiS and BiL), brachioradialis (Brd), flexor capri radialis (FCR), flexor carpi ulnaris (FCU), and extensor capri radialis (ECR). EMG recordings were temporally synchronized with the motion capture and virtual reality systems during reaching as described in Talkington *et al*. [14].

### Data Analysis

Motion capture data were low pass filtered at 10 Hz and interpolated with a cubic spline. Arm kinematics was obtained from motion capture by defining local coordinate systems representing the trunk, arm, forearm, and hand. Joint angles representing 5 DOFs of the arm were derived using linear algebra, namely shoulder flexion-extension, shoulder abduction-adduction, shoulder internal-external rotation, elbow flexion-extension, forearm pronation-supination, and wrist flexion-extension (Fig. 1A, example for one DOF). Active muscle torques and their components were calculated from kinematic data using a dynamic model of the arm with 5 DOFs (Simulink, MathWorks) as described in [12]. The individual height, weight, and segment lengths together with the published anthropometric proportions were used to scale the model inertia to individual body sizes [15]. Inverse simulations were run with the angular kinematics as input and the active torques produced by muscles, termed muscle torques (MT), as output. Simulations were ran with and without gravity force to estimate the components of muscle torque responsible for supporting the arm against gravity (postural component of muscle torque or MT_P_) and for intersegmental dynamics compensation (dynamic component of muscle torque or MT_D_) as described in detail in Olesh *et al*. [12]. The simulations were run for each trial. Torque profiles were normalized in time, averaged per movement direction per DOF and per subject, and down sampled to 100 samples (Fig. 1B, example for one DOF). Maximum torque amplitudes were determined per DOF per participant’s arm and used to scale the amplitudes of averaged profiles across movement directions and workspaces.

EMG recordings were high-pass filtered at 20 Hz to remove motion artifacts, rectified, and low-pass filtered at 10 Hz [16]. Next, EMG recordings were normalized to movement duration, down sampled to 100 samples, and averaged per movement direction. Maximum contraction values were determined for each muscle and participant across averaged EMG timeseries per movement direction and used to scale the amplitudes of averaged profiles (Fig. 1C).

**Fig 2.**
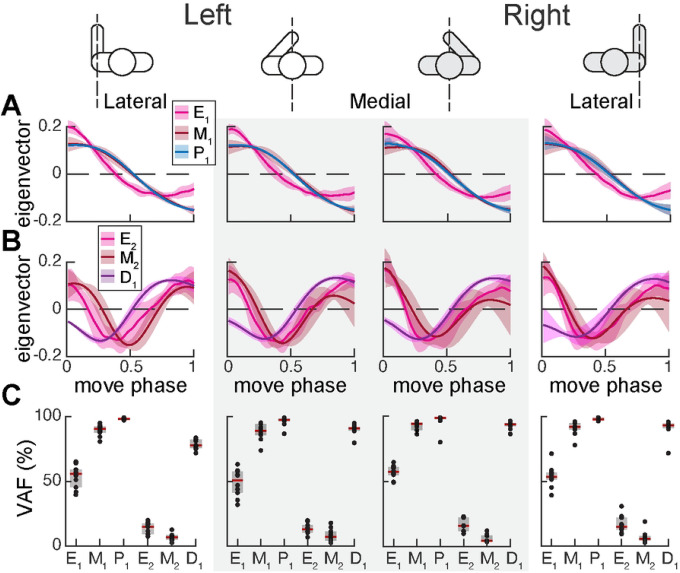
Principal components of EMG and muscle torques. Data from the four conditions indicated by the pictograms are shown in plots arranged in columns. The data is from 10 participants who performed all 4 conditions. (**A**) Solid lines show profiles of the 1^st^ principal component obtained from EMG (E_1_), muscle torque (M_1_), and the postural component of muscle torque (P_1_). (**B**) Solid lines show profiles of the 2^nd^ principal component obtained from EMG (E_2_) and muscle torque (M_2_), and the 1^st^ principal component obtained from the dynamic component of muscle torque (D_1_). (C) Variance accounted for (VAF) by principal components obtained from EMG and muscle torques.

Principal Component Analysis (PCA) was used to reduce the dimensionality of EMG and torque data similar to Olesh *et al*. [12]. Normalized and demeaned EMG profiles from 12 muscles of 28 center-out reaching movements (14 center-out and 14 return movements between each pair of targets) were combined into one matrix, ***A***_***n×m***_, where n = 100 (time) and m = 336 (movement directions × muscle). PCA was applied to the data from each of the four conditions (LLat, RLat, LMed, and RMed) separately. Thus, four ***A***_***n×m***_ matrices were obtained for each subject. Similarly, normalized and demeaned muscle torque profiles (MT, MT_P_, and MT_D_) from 5 DOFs of 28 center-out reaching movements were combined into one matrix, ***B***_***n×m***_, where n = 100 (time) and m = 140 (movement directions × DOF). Since the analysis included three types of torques (MT, MT_P,_ MT_F_) in the four conditions, twelve ***B***_***n×m***_ matrices were obtained for each subject. PCA was performed using *pca* function from MATLAB Statistics and Machine Learning Toolbox. Each matrix was centered, and a default singular value decomposition algorithm was applied to obtain principal component eigenvectors (Fig. 2A, B), the variance accounted for (VAF) by each principal component (Fig. 2C), and scores that are the representations of the matrix in the principal component space. It is important to note that reaching in the same direction leftward or rightward from the starting location rely on different muscles due to the bilateral symmetry of our body. That is why the reaches that include leftward movements to the targets on the left from the central target performed by the left hand were compared to the mirror rightward movements to the right from the central target performed by the right hand and vice versa.

### Statistics

To test the hypotheses, we performed two types of statistical tests. The first test compared the profiles of the 1^st^ and 2^nd^ principal components obtained from EMG to the principal components obtained from muscle torques using a correlation analysis (*corr* function in MATLAB Signal Processing Toolbox). We used repeated measures analysis of variance (RM ANOVA, *ranova* function in MATLAB Statistics Toolbox) to test the null hypotheses that the coefficients of determination (R^2^) from all conditions and data types come from the same distribution. Post-hoc multiple comparisons were done using *multcompare* function in MATLAB Statistics Toolbox. We applied Bonferroni-Sidak correction to correct the alpha for familywise error, so that the adjusted alpha for the comparison between 6 combinations between 4 conditions = 0.0085, for the comparison between 7 meaningful combinations of two principal components = 0.0073.

The second test evaluated how muscles co-activate across changing starting postures and movement directions. The rationale is that reaching in different directions is caused by different amplitudes of postural or dynamic muscle torques. Therefore, the muscles that cause the actions in the appropriate directions will coactivate together in proportion to the difference in forces between movements. This implies that there should be a correlation between the activation amplitude of two muscles that work together across multiple movement directions, i.e., classical muscle synergies should emerge. To test this idea, we have utilized a linear correlation analysis (*corr* and *corrplot* functions in MATLAB Statistics and Econometrics Toolboxes) to quantify the linear relationships between principal component scores from pairs of muscles across movements. First, we extracted the scores of the 1^st^ principal component from EMG and concatenated them into matrix ***C***_***n×m***_, where n = 28 (movement directions) and m = 12 (muscles) per condition per subject. Then we evaluated the statistical significance of the linear relationships between scores of 66 muscle pairs (Fig. 4). We applied Bonferroni-Sidak correction to correct the alpha for familywise error, so that the adjusted alpha = 0.0008. We repeated this analysis for the 2^nd^ principal component from EMG.

Four older adults out of the total of 14 participants did not complete the reaching in the medial workspace (RMed and LMed). Therefore, the data from 10 adults (9 young adults, and one older adult; mean age = 26.3 ± 11.2 years) who completed all 4 tasks are reported here. The same statistical analysis was performed on all participants across the two workspace s and reported in Results. Supplementary figures show the results of the same analysis with inclusion of all individuals across the LLat and RLat tasks (Supplementary Figures S1 and S2).

## Results

All participants were able to complete the center out task with either left or right arms with consistent kinematics (Fig. 1A). We expected that movements with left and right arms with the same starting positions and kinematics in forward, backward, up, or down directions would rely on similar forces produced by the same muscles. These forces are captured here with muscle torques that are further subdivided into a linear combination of postural and dynamic components. The muscle torque profiles accompanying the corresponding movements had very similar trajectories across conditions (Fig. 1B). The were also static offsets in the postural components of muscle torques underlying matching movement directions. For example, holding the initial position at the beginning of the movement required larger muscle torques about the right shoulder flexion/extension DOF (RMed and RLat conditions) compared to that for the left shoulder in matching conditions (LMed and LLat conditions respectively, Fig. 1B). This is likely due to the motor redundancy, i.e., the different combinations of joint angles and torques that can result in the same position of the hand. From our earlier work we knew that important features of muscle activity can be captured by the postural and dynamic components of muscle torques [12]. However, in that study we focused our analysis on the movements performed by the dominant arm in the lateral workspace. Here the analysis encompasses different workspaces and both arms, which enables us to evaluate the generalizability of this conclusion. We found that intra- and inter-subject variability of EMG profiles were very low. The average standard deviation of EMG profiles across movement repetitions for muscles in the dominant right arm (normalized to maximal EMG of each muscle) was 12% ± 1% across 14 subjects. For the muscles in the non-dominant left arm the inter-trial variability was 22% ± 2% across 14 subjects. This shows that the muscle activation profiles were more consistent across repetitions of the same movement type for the dominant arm compared to the non-dominant arm. Across 14 subjects, the average normalized standard deviation of EMG profiles of muscles in the dominant right arm was 11% ± 4%. For the muscles in the non-dominant arm the inter-subject variability was 11% of maximal EMG ± 3%. Moreover, the EMG profiles also broadly reflected the differences in the joint torques across conditions (Fig. 1C). In the example for the reach forwards and up in Fig. 1, the amplitude of anterior deltoid activity increased in parallel with the increases in the postural component of the shoulder flexion muscle torque across conditions (Fig. 1, MT_P_ and ADel). Furthermore, the initial propulsive flexion moment in the dynamic muscle torque profile was preceded by a burst in the activation of the long head of biceps (Fig. 1, MT_D_ and BiL, end of the burst is visualized). However, EMG profiles are notoriously noisy and difficult to interpret. Therefore, in the following analysis we will broadly test the generalizability of conclusions from Olesh et al. [12] by focusing on the salient features in EMG obtained with PCA as described in Methods. To test the hypotheses, we will compare those salient features in EMG with the corresponding features in muscle torques.

The temporal profile of the 1^st^ principal component (eigenvector E_1_) obtained from EMG matched the 1^st^ principal component (M_1_) obtained from muscle torques (MT) and the 1^st^ principal component (P_1_) obtained from the postural components only of the muscle torques (MT_P_; Fig 2A). This feature captures the posture-related changes in EMG that underly gradually increasing or decreasing EMG profiles. The temporal profile of the 2^nd^ principal component (E_2_) obtained from EMG matched the 2^nd^ principal component (M_2_) obtained from muscle torques and the 1^st^ principal component (D_1_) obtained from the dynamic components only of the muscle torques (MT_D_) although with a phase shift (Fig 2B). This feature captures the focal movement-related changes in EMG that underly bursts in EMG profiles. The VAF by the 1^st^ principal components obtained from muscle torques (M_1_, P_1_, and D_1_) was very high (Fig. 2C), the mean values across conditions and all participants were 90% for M_1_, 97% for P_1_, and 88% for D_1_ (Table 1 contains values per condition). This shows that the profile of muscle torques was very similar across joints and DOFs, indicating a high degree of intralimb coordination. P_1_ captured more variance across conditions and DOFs than M_1,_ similarly D_1_ captured more variance across conditions and DOFs than M_2_ despite similar profiles. This shows that subdividing muscle torques into postural and dynamic components results in more reliable features compared to those obtained with PCA of muscle torques. As expected, the EMG VAF by the 1^st^ principal component was lower than the muscle torque VAF by the 1^st^ principal component due to higher noise in EMG data (Fig. 2C), the mean VAF across conditions and participants was 53% for E_1_, but the opposite was true for the 2^nd^ principal component: mean VAF by E_2_ was 15% while the mean VAF for M_2_ was 7%. Moreover, both EMG and muscle torque VAF by the corresponding principal components were similar across limbs and workspace s (Fig. 2C). This indicates that highly stereotypical muscle torques during reaching movements explain a high portion of the overall variance in EMG profiles in multiple arm muscles. The same conclusion was reached in Olesh et al. [12] from a smaller subset of data. Moreover, our results further support the idea that E_1_ captures the muscle recruitment necessary for generating postural forces to support the arm against gravity, while E_2_ captures the muscle recruitment necessary for generating dynamic forces for propulsion during acceleration and deceleration phases of reach. This supports the first hypothesis that the muscle activity profiles reflect the forces needed to produce the movement.

**Fig 3.**
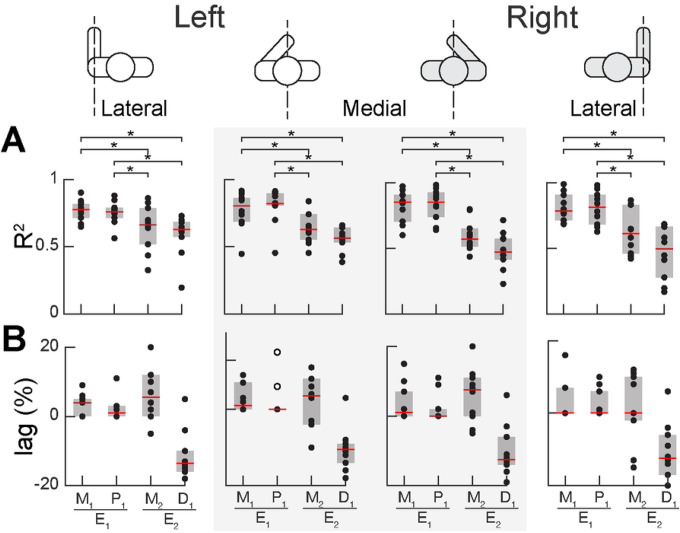
Correlations between the temporal profiles of principal components derived from EMG and muscle torques. Data (N=10) from the four conditions indicated by the pictograms are shown in plots arranged in columns. (A) Dots show individual R^2^ values from cross-correlations between profiles, red lines show median values, and grey boxes show interquartile ranges. Brackets with stars indicate significant relationships with correction for familywise error. (B) Dots show individual lag values from cross correlations, red lines show median values, and grey boxes show interquartile ranges. Open dots are outliers. E_1_ and E_2_ are the 1^st^ and 2^nd^ principal components respectively obtained from EMG; M_1_ and M_2_ are the 1^st^ and 2^nd^ principal components respectively obtained from muscle torques; P_1_ and D_1_ are the 1^st^ principal components obtained from the postural and dynamic components of muscle torque respectively.

The degree of similarity between features obtained with PCA was compared statistically using cross correlation analysis as described in Methods. RM ANOVA on the coefficients of determination (R^2^) between principal component profiles showed a significant main effect F(15, 120) = 11.39, *p* = 0.00. Post-hoc tests have shown that there were no differences between the R^2^ values across conditions (Table 2) and that the temporal profiles of E_1_, M_1_, and P_1_ were more similar to each other than to E_2_, M_2_, or D_1_ and vice versa (Fig. 3A; Table 3). Similar results were observed when data from all 14 individuals who performed reaches from only the lateral workspace were analyzed (Supplementary Fig. S1). The RM ANOVA main effect was significant (F(7, 84) = 12.62, *p* = 0.00) and the post-hoc test did not reject the null hypothesis that the data from LLat and RLat conditions comes from the same distribution (MSE = 0.02 ± SE 0.02; p = 0.41). The temporal profiles of E_1_, M_1_, and P_1_ were more similar to each other than to E_2_, M_2_, or D_1_ and vice versa (Supplementary Fig. S1A; Table 4). This further supports our first hypothesis and the idea that the first and second principal components of EMG represent postural and dynamic forces. These conclusions generalize across both limbs and workspace s. This may be useful for removing noise from muscle activity signals and increasing the interpretability of surface EMG recordings.

Additionally, we observed a consistent phase lead of the D_1_ components onto the E_2_ component (Fig. 3B, Supplementary Fig. S1B). This phase lead can potentially be taken advantage of to predict the changes in the underlying muscle forces or contractions from dynamical simulations prior to observable motion and improve performance of real-time control applications.

**Fig 4.**
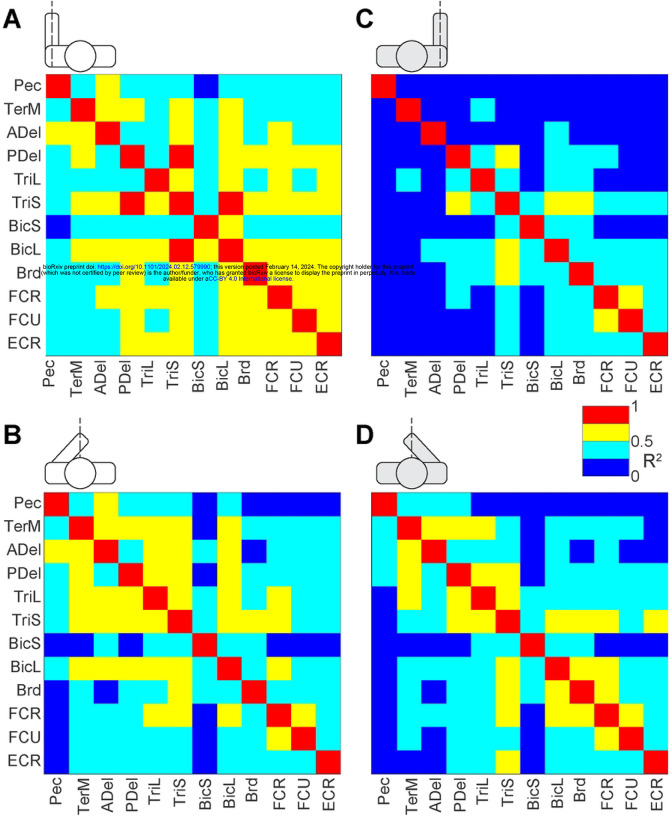
Example linear regressions between scores across reaching directions. Example regression matrix between E_1_ scores of 12 muscles across 28 right-handed reaches within the medial workspace (RMed condition) for a single participant. The coordinates of each dot represent principal component scores for two muscles for a single movement direction. Histograms show the distribution of the scores for a given muscle across reaching directions. Solid lines show least-squares linear regression, red lines indicate significant relationships with correction for familywise error, so that the adjusted alpha = 0.0008.

**Fig 5.**
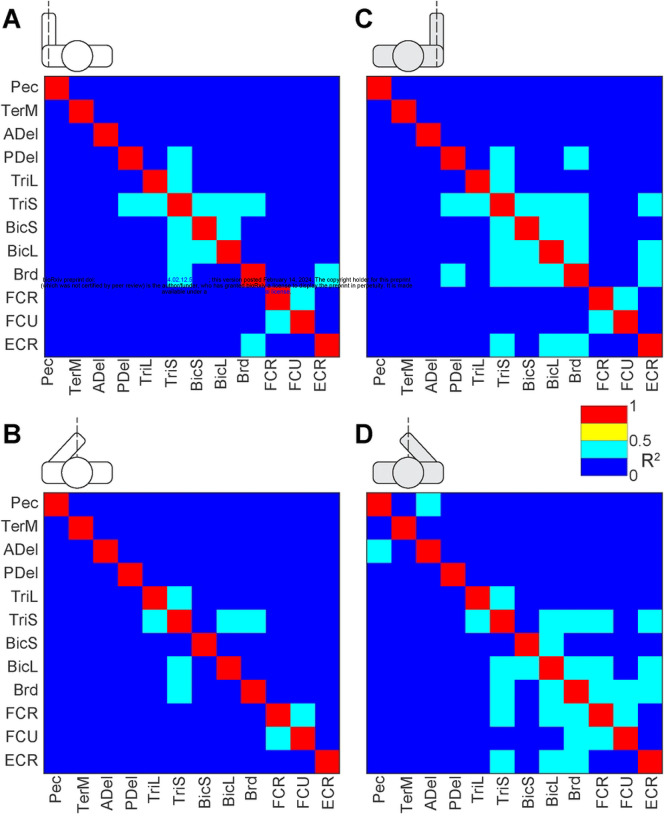
Posture-related muscle coactivation across all conditions. Heatmaps show coefficients of determination (R^2^) from regressions shown in Fig. 4 averaged across 10 participants. Blue colors represent minor or no significant relationships between E_1_ coefficients across movement directions, while red and yellow colors represent moderate and strong relationships. Pictograms indicate conditions, i.e., LLat (A), LMed (B), RLat (C), and RMed (D). Muscles are abbreviated as described in Methods.

**Fig 6.**
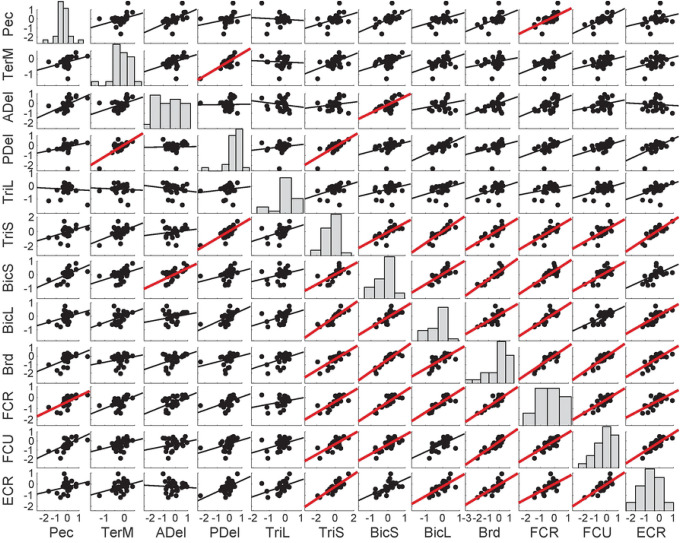
Propulsion-related muscle coactivation across all conditions. Heatmaps show coefficients of determination (R^2^) from regressions shown in Fig. 4 averaged across 10 participants. Blue colors represent minor or no significant relationships between E_2_ coefficients across movement directions, while red and yellow colors represent moderate and strong relationships. Pictograms indicate conditions, i.e., LLat (A), LMed (B), RLat (C), and RMed (D). Muscles are abbreviated as described in Methods.

The second hypothesis was that muscle synergies captured with dimensionality reduction methods represent force directions controlled by the CNS. The rationale for the test of this hypothesis is that the synergies must reflect the dynamic requirements of each reaching movement. This means that for movements requiring a larger change in the postural moments, a consistent group of muscles will have higher scores for the principal component representing that synergy (E_1_) in the corresponding movement and the same group of muscles will have lower scores in other movements with smaller changes in postural moments. The same logic applies to the synergy responsible for the generation of dynamic forces (E_2_). This means that if the primary features in EMG (E_1_ and E_2_) represent muscle synergies, we expect significant linear correlations between scores of pairs of muscles across movement directions. We performed this linear correlation analysis by comparing scores between pairs of muscle across reaching in multiple directions (Fig. 4 shown an example for one condition and participant). The scores represent the projection of EMG onto the principal component vectors. When the scores are close to 0, this means that the components do not capture much variance in the corresponding EMG profile. For all combinations of muscles, movements, and participants, about 21% of the E_1_ scores were low, i.e., values were below 5% of maximal score (RLat: 25%, LLat: 18%, LMed: 16%, and RMed 24%). About 26% of E_2_ scores were low (RLat: 27%, LLat: 26%, LMed: 22%, and RMed: 28%). The low scores are likely noise. Therefore, no relationships between the low score values are expected, as seen for example in the insignificant correlation between low scores of BicL and ADel in the example shown in Fig. 4. Despite the noise, we observed multiple correlations between E_1_ scores in certain conditions. For reaching within the left lateral workspace, 68% of muscles (45 muscle pairs out of 66) in the non-dominant arm showed moderate correlations between their E_1_ scores (R^2^ ≥ 0.5; Fig. 5A). For reaching within the medial workspace, 35% of muscles (23/66 pairs) in the non-dominant arm showed moderate correlations between their E_1_ scores (Fig. 5B). In contrast, only 12% of muscles in the dominant arm showed moderate correlations when reaching within the right lateral workspace (Fig. 5C). For reaching within the shared medial workspace, 32% of muscles in the dominant arm showed moderate correlations (Fig. 5D), similar to that for the non-dominant arm (Fig. 5B). Overall, more correlations were observed between E_1_ scores in muscles of the non-dominant arm. There were fewer muscle pairs with consistently correlated E_2_ scores (Fig. 6). The correlations between E_2_ scores were much lower, so that on average none of the muscle pairs showed moderate correlations across all 4 conditions. However, despite the higher percentage of low E_2_ scores, there were significant correlations between select muscle pairs (see below) with more correlations occurring between E_2_ scores in muscles of the dominant arm compared to the non-dominant arm, in contrast to the correlations between E_1_ scores. The same results were obtained from the data across 14 participants who performed the RLat and LLat conditions only (Supplementary Figures S2). The larger number of subjects included in the analysis across lateral workspaces reduced the number of significant correlations between E_1_ scores in the muscles of the non-dominant arm only (Fig. 5A vs. Supplementary Figure S2A). Overall, these results reject the second hypothesis that specific force directions are controlled through muscle synergies.

Correlations between scores were overwhelmingly positive, indicating that not only agonistic but also antagonistic muscles, such as biceps and triceps, changed their activity together. The coactivation between muscles of the non-dominant arm associated with counteracting gravity load on the limb (correlations between E_1_ scores) was the broadest in the LLat condition. Two salient muscle groups comprised primarily the proximal muscles (TerM, ADel, PDel, TriL, and TriS) and distal muscles (BiL, Brd, FCR, FCU, and ECR), both highly coupled (Fig. 5A). In contrast, the coactivation between muscles of the dominant arm in RLat condition consisted of three much smaller muscle groups (PDel/TriS, TriS/BicL/Brd, and FCR/FCU) with only the first two groups coupled (Fig. 5C). These three small clusters were the only consistent groups of coactivating muscles across both workspaces and both limbs. For forces needed to propel the arm, the E_2_ scores for three pairs of muscles of the dominant arm positively correlated for reaching in the lateral workspace (R^2^ for BicL/TriS = 0.49, Brd/TriS = 0.45, and Brd/BicL = 0.45). There were 6 significantly correlated muscle pairs in RLat condition that were similar across at least 5/10 subjects (Brd/ECR, FCR/FCU, Brd/BicL, Brd/TriS, BicL/BicS, and BicL/TriS) and 4 significantly correlated muscle pairs in RMed condition (Brd/ECR, FCR/FCU, Brd/TriS, and TriS/TriL). For the non-dominant arm, there were 2 significantly correlated muscle pairs in LLat condition that were similar across 6/10 subjects (Brd/TriS and BicL/TriS) and 1 significantly correlated muscle pair in LMed condition (BicL/TriS). Note that in these reaching movements with the arm pronated, the muscles performing the antigravity action would be ADel, BiL, Brd, and ECR, while the rest would produce joint moments in the direction of gravity. Here we observed consistent coactivation of not only antigravity agonists, but also antagonists, such as biceps and triceps. Such coactivation of antagonistic muscles modulates joint stiffness, suggesting that this may be the control strategy for supporting the body against gravity.

## Discussion

We observed that highly stereotypical muscle torques during reaching movements explain a high portion of the overall variance in EMG profiles in multiple arm muscles. We have shown that the first principal component of EMG captures the muscle recruitment necessary for generating postural forces to support the arm against gravity, while the second principal component of EMG captures the muscle recruitment necessary for generating dynamic forces for propulsion during acceleration and deceleration phases of reach (Fig. 2, 3, Supplementary Fig. S1). This supports our first hypothesis showing that the first principal components of EMG represent postural and dynamic forces. This shows that subdividing muscle torques into postural and dynamic components captures muscle contributions to postural and propulsive forces. These conclusions, originally derived from a smaller subset of movements by the dominant arm in one area of reaching workspace [12], are generalizable to movements by the non-dominant arm and across a larger reaching workspace that overlaps between both arm arms. This may be useful for removing noise from muscle activity signals and increasing the interpretability of surface EMG recordings.

Moreover, we observed biomechanically expected correlations between scores of muscle pairs across movements in very few conditions (Fig. 5, 6, Supplementary Fig. S2). This shows that when joint torques in specific directions (flexion or extension) were required to reach toward a target, muscles were *not* recruited in consistent groups in most conditions. This result rejects the second hypothesis that specific force directions are controlled through muscle synergies. When correlations between scores were observed when producing postural forces by the non-dominant arm, they were overwhelmingly positive, despite the antagonistic actions between multiple muscle pairs in our dataset. This suggests that the arm is supported against gravity not through the production of reaction forces with antigravity muscles, but rather through the modulation of limb stiffness.

It is possible that the reason we have not obtained consistent muscle synergies across movements is that surface EMG was too noisy and variable across the different movements and individuals. However, our variability data reported in Results shows that the temporal profiles of EMG were very consistent across all these conditions, especially for the dominant arm with the fewest observed coactivations. Another evidence of the consistency of EMG profiles is the large percentage of variance that is captured in EMG by the first two principal components (Fig. 2) and by the components obtained from muscle torques (Fig. 3), which are highly stereotypical during reaching movements. Therefore, it seems highly unlikely that the inter-movement or inter-subject variability can explain the lack of consistent coactivation of muscle pairs for the production of joint torque in a given direction in most conditions.

Muscle synergies are often proposed as a method of reducing the dimensionality of neural control space and overcoming the redundancy of the musculoskeletal system [4–8]. We have posited that it is not sufficient to show the existence of a low dimensional space, one needs to demonstrate the utility of the obtained synergies in performing specific movements. Here we define muscle synergies as groups of muscles controlled with a single control signal that produces joint torque about one or mode DOFs in a specific direction. This definition is similar to force fields first defined by Bizzi [17]. This definition implies that when movements require a force vector in the same direction but of different amplitude, the recruitment level of the muscle synergy needed to produce that force vector should also vary in scale with the amplitude of the force vector across those movements. In other words, if a simple synergy exists for a single input recruiting both biceps and brachioradialis to produce elbow flexion torque, then these muscles should activate together less in movements needing lower elbow flexion torque and more in movements needing higher elbow flexion torque. This logic is what motivated our second hypothesis, which we tested using the regression analysis shown in Fig. 4. The level of coactivation of muscle synergies was captured by the PCA scores. Therefore, examining changes in PCA scores across different movement directions, i.e. across different force directions underlying these movements, reveals the pattern of synergistic coactivation of muscles. Surprisingly, we found consistent coactivation of very few pairs of muscles and primarily in the component of muscle activation responsible for overcoming gravity (Fig. 5, Supplementary Fig. 2A, B). Limited consistent coactivations were observed in the phasic component of distal muscles responsible for acceleration/deceleration forces (Fig. 6, Supplementary Fig. 2C, D). This means that when joint moments in the same direction but different amplitudes need to be produced for movements toward different targets, it is accomplished by activation of different combinations of muscles requiring separate neural recruitment signals. These results contradict the idea of muscle synergies driving specific force directions and lead us to reject the second hypothesis.

Our results support the idea of internal models embedding body dynamics. Internal models are defined as neural networks in the brain that simulate the dynamics of the body and its interaction with the external world [1,3,18–21]. Forward models predict the consequences of a given action, for example, predicting sensory feedback without delay from an efferent copy of a corticospinal motor command [22,23]. Inverse models capture information in the opposite direction [3,24]. They convert a motor plan into the motor execution output of the motor system that converges on the motoneurons in the spinal cord. The motor plan is thought to be developed in the premotor areas, where neural activity has many kinematic features in the extrinsic head-centered reference frame resolving the kinematic redundancy problem [25–27]. The motor execution output is thought to originate in the primary motor cortex, where neural activity has many dynamic features in the intrinsic reference frame [28–30]. The transformations from motor plan into motor execution may potentially resolve the muscle redundancy problem by embedding biomechanical constraints. These constraints may take the form of embedded muscle anatomy relationships, such as known ratios between moments arms of muscles about a given DOF [31].

Reaching movements are often separated into two distinct actions 1) the transport action that involves gross stereotypical movement of the whole arm to transport and orient the hand and 2) the manipulation action that involves fine movements of the hand. This separation is reflected in the anatomical organization of muscles that form *proximal* and *distal* groups that could serve as *separate control targets* [9]. Interestingly, we observed here distinct correlations between the principal component scores of proximal and distal muscle pairs, particularly in the non-dominant arm for the gross postural component and in the dominant arm for the propulsive component. The proximal group of muscles including deltoids, biceps short head, and both heads of triceps, co-activated in proportion to the gravity-related forces acting on the limb (top left corners of correlation matrices in Fig. 5A, B and Supplementary Fig. S2A). The distal group of muscles included wrist flexor muscles and brachioradialis, it co-activated to a lesser degree in proportion to the propulsion-related forces acting on the limb, sometimes including the long head of biceps or the short head of triceps (bottom right corners of correlation matrices in Fig. 5, 6, and Supplementary Fig. S2). These clusters of proximal and distal muscle coactivations suggests that there may be separate control signals responsible for activating proximal and distal musculature, which are coordinated together when wrist stabilization is required. Altogether, this evidence supports the idea of two interacting neural controllers targeting proximal and distal muscle groups in the arm and hand performing gross postural and fine goal-directed motor control during reaching [32]. This idea is supported by the neural stimulation experiments showing that cortical muscle representations are organized somatotopically based on their proximal-to-distal anatomy, the famous motor homunculus [33,34]. Non-invasive stimulation of human motor cortex can facilitate differentially proximal and distal muscles depending upon the demands of a particular task, i.e., reaching vs. grasping [35]. Distinct white matter tracts demonstrate some preferential contribution for proximally-controlled gross movements versus distally-controlled fine motor skills [36]. The proximal controller may be more energetically favorable for controlling whole arm impedance to support the limb against gravity [37]. The distal controller may be more attuned to stabilizing the hand during transport and compensating for forces from external objects during their manipulation. The proposed proximal and distal controllers may also underlie the differences in controlling limb dynamics and adaptation to novel limb dynamics between dominant from nondominant arms [38,39]. Overall, our data supports the idea of two neural controllers that target different muscle groups in the arm and hand for gross postural and fine goal-directed control of reaching.

## Figures and Tables

**Table 1.** Variance accounted for across reaching conditions.

	Left lateral	Left medial	Right medial	Right lateral	Total
E_1_	53.46 ± 8.83	48.88 ±10.51	57.05 ±5.05	53.91 ±8.43	53.33 ± 8.21
M_1_	89.51 ± 4.35	88.33 ± 6.55	92.35 ± 3.77	91.04 ± 5.33	90.31 ± 5.00
P_1_	98.09 ± 0.73	96.46 ± 3.70	96.63 ± 5.88	98.19 ± 1.02	97.34 ± 2.83
E_2_	13.90 ± 4.58	13.39 ± 4.11	15.84 ± 4.93	16.79 ± 6.70	14.98 ± 5.08
M_2_	6.99 ± 2.81	8.43 ± 4.93	5.49 ± 3.04	6.94 ± 4.67	6.96 ± 3.86
D_1_	78.39 ± 3.83	90.25 ± 4.08	92.99 ± 2.85	91.27 ± 7.00	88.23 ± 4.44

Values are means ± standard deviation across 10 individuals.

**Table 2.** Post-hoc comparisons of Pearson’s correlation coefficients across conditions.

Condition 1	Condition 2	MSE ± SE	*p* value
Llat	Lmed	−0.02 ± 0.03	0.95
Llat	Rlat	0.02 ± 0.03	0.91
Llat	Rmed	0.01 ± 0.03	0.99
Lmed	Rlat	0.03 ± 0.04	0.82
Lmed	Rmed	0.02 ± 0.03	0.88
Rlat	Rmed	−0.01 ± 0.02	0.95

MSE – mean squared error, SE – standard error of the mean. Adjusted alpha = 0.0085.

**Table 3.** Post-hoc comparisons of Pearson’s correlation coefficients between EMG- and torque-based components across four conditions and 10 participants.

Condition 1	Condition 2	MSE ± SE	*p* value
E_1_/M_1_	E_1_/P_1_	−0.01 ± 0.01	0.7739
E_1_/M_1_	E_2_/M_2_	0.19 ± 0.03	**0.0018**
E_1_/M_1_	E_2_/D_1_	0.29 ± 0.05	**0.0014**
E_1_/P_1_	E_2_/M_2_	0.20 ± 0.04	**0.0068**
E_1_/P_1_	E_2_/D_1_	0.31 ± 0.06	**0.0069**
E_2_/M_2_	E_1_/P_1_	−0.20 ± 0.04	**0.0069**
E_2_/M_2_	E_2_/D_1_	−0.10 ± 0.04	0.1772

MSE – mean squared error, SE – standard error of the mean. Bold *p* values show significant difference with adjusted alpha = 0.0073.

**Table 4.** Post-hoc comparisons of Pearson’s correlation coefficients between EMG- and torque-based components across two conditions and 14 participants.

Condition 1	Condition 2	MSE ± SE	*p* value
E_1_/M_1_	E_1_/P_1_	0.00 ± 0.01	0.9999
E_1_/M_1_	E_2_/M_2_	0.15 ± 0.03	**0.0006**
E_1_/M_1_	E_2_/D_1_	0.23 ± 0.05	**0.0016**
E_1_/P_1_	E_2_/M_2_	0.15 ± 0.04	**0.0044**
E_1_/P_1_	E_2_/D_1_	0.23 ± 0.05	**0.0028**
E_2_/M_2_	E_1_/P_1_	−0.15 ± 0.04	**0.0044**
E_2_/M_2_	E_2_/D_1_	−0.08 ± 0.04	0.2435

MSE – mean squared error, SE – standard error of the mean. Bold *p* values show significant difference with adjusted alpha = 0.0073.
